# Mobile Measurements of Particulate Matter in a Car Cabin: Local Variations, Contrasting Data from Mobile versus Stationary Measurements and the Effect of an Opened versus a Closed Window

**DOI:** 10.3390/ijerph15122642

**Published:** 2018-11-26

**Authors:** Janis Dröge, Ruth Müller, Cristian Scutaru, Markus Braun, David A. Groneberg

**Affiliations:** 1 Institute of Occupational, Social, and Environmental Medicine, Goethe University Frankfurt, Theodor-Stern-Kai 7, 60590 Frankfurt am Main, Germany; ruth.mueller@med.uni-frankfurt.de (R.M.); m.braun@med.uni-frankfurt.de (M.B.); groneberg@med.uni-frankfurt.de (D.A.G.); 2 Institute of Occupational Medicine, Charité–School of Medicine, Humboldt-University & Free University, Augustenburger Platz 1, 13353 Berlin, Germany; Cristian.Scutaru@charite.de

**Keywords:** particulate matter, mobile air quality study, in-cabin exposure, particle size distribution, traffic emissions, ventilation modes

## Abstract

Air pollution of particulate matter (PM) from traffic emissions has a significant impact on human health. Risk assessments for different traffic participants are often performed on the basis of data from local air quality monitoring stations. Numerous studies demonstrated the limitation of this approach. To assess the risk of PM exposure to a car driver more realistically, we measure the exposure to PM in a car cabin with a mobile aerosol spectrometer in Frankfurt am Main under different settings (local variations, opened versus a closed window) and compare it with data from stationary measurement. A video camera monitored the surroundings for potential PM source detection. In-cabin concentrations peaked at 508 µg m^−3^ for PM_10_, 133.9 µg m^−3^ for PM_2.5_, and 401.3 µg m^−3^ for coarse particles, and strongly depended on PM size and PM concentration in ambient air. The concentration of smaller particles showed low fluctuations, but the concentration of coarse particles showed high fluctuations with maximum values on busy roads. Several of these concentration peaks were assigned to the corresponding sources with characteristic particle size distribution profiles. The closure of the car window reduced the exposure to PM, and in particular to coarse particles. The mobile measured PM values differed significantly from stationary PM measures, although good correlations were computed for finer particles. Mobile rather than stationary measurements are essential to assess the risk of PM exposure for car passengers.

## 1. Introduction

Urban agglomeration areas are usually characterized by a high rate of air pollution caused by particulate matter (PM). In urban areas, PM is mainly emitted by cars burning fossil fuel or during abrasion processes of vehicle tires and brakes, respectively, from raised dust. Further sources are power plants and chemical or metallurgical factories [1,2]. Very high concentrations can especially be reached nearby roads [3]. PM can contain particles with a lot of different sizes [4]. For monitoring air quality, usually the particle fractions PM_10_ (particulate matter with an aerodynamic diameter less than 10 µm), PM_2.5_ (particulate matter with an aerodynamic diameter less than 2.5 µm), and PM_1_ (particulate matter with an aerodynamic diameter less than 1 µm) are used. Particles with an aerodynamic diameter between 2.5 and 10 µm belong to coarse fraction (PM_coarse_) [5]. High exposures of PM have negative consequences for human health and are therefore a key issue in current research [6]. Already, very low concentrations of PM can increase the blood pressure or raise the risk of cardiovascular diseases [7]. Recent research showed that even short-term exposures with very high PM concentrations, which are typical for traffic participants, can be harmful to health [8,9,10]. Under long-term exposure, the consequences can be even worse. The probability for the formation of lung carcinomas increases [11]. A long-term exposure can also negatively affect fetal growth or changes heart rate variability [12,13,14,15,16]. Due to increased health risks, especially in urban areas, where anthropogenic emissions of PM are the highest, further research is obligatory. Many cities have a system of air quality monitoring stations (AQMS) for getting an overview of the local PM pollution [17]. The AQMS data is often used to estimate the PM exposure for society. Based on this knowledge, guidelines are created and political decisions are made [18]. Because AQMS measurements are only for outside air and the PM concentration with its respective sources is generally distributed very heterogeneously within a certain area, it is questionable whether these static measurements are generally suitable for estimating the actual exposure to traffic participants, especially to passengers inside a car [19,20,21,22]. In Germany, for example, people spend, on average, 1 h and 28 min a day travelling, depending on their occupation and residence, mainly in cars [23]. Some occupation groups, such as taxi or courier drivers, spend even more time in their cars. During that driving time, most of the daily PM uptake is made [24,25,26]. Because of such a high medical relevance, monitoring PM concentrations in the driver cabin is an essential research field. Frankfurt am Main, the study site in the presented study, is predestinated for a mobile air quality study because of its extremely high commuter traffic and a population density of almost 3000 people/km². This value is one of the highest in Germany [27]. In the course of this study, local and temporal variations of PM concentrations and particle sizes in the cabin of a car were characterized while driving on an urban circular drive in Frankfurt am Main. Furthermore, the PM concentration and the particle size distribution from rides with an opened window (OW) and closed window (CW) have been compared with each other. By comparing the measured data in a car cabin with AQMS data, the suitability of the AQMS data for estimating the actual PM exposure for a driver was tested to assess his potential health risk from exposure to traffic-related PM.

## 2. Materials and Methods

### 2.1. Measurement of Particulate Matter

**Figure 1:**
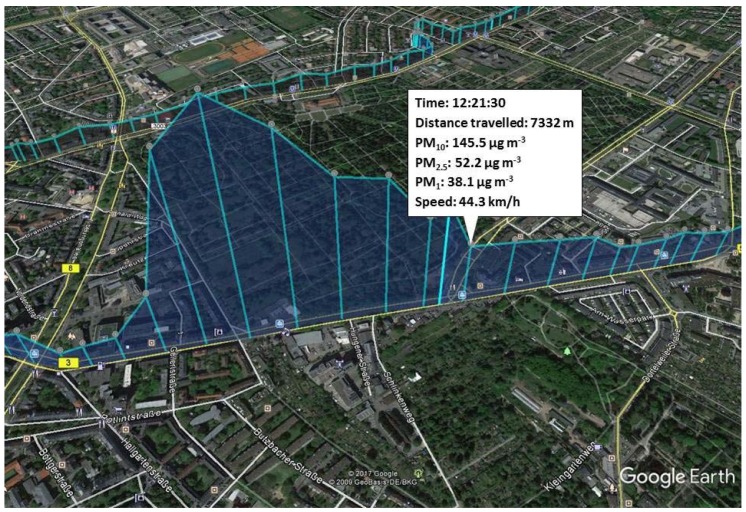
kml file with the local in-cabin particulate matter (PM) concentration measured while driving through Frankfurt am Main. Turquoise line: Location-dependent PM concentration. Geographical picture by Google Earth.

Car rides for the mobile air quality study were made with an electric vehicle of the BMW i3 model with the intention to avoid an exhaust related increase of in-cabin PM concentration by the car itself. The PM in-cabin concentration was measured by an aerosol spectrometer, type GRIMM 11-R. It was used according to the quality guidelines of the supplier and calibrated before the measurement. The suction of the instrument was placed at the co-driver’s seat at head height to get a realistic image of the actual PM exposure to the driver in the cabin. The spectrometer was connected to a laptop (Fujitsu Lifebook) and a GPS (Global Positioning System) mouse. Every six seconds, a value was generated for the fractions, PM_10_, PM_2.5_, and PM_1_. The measured PM data was linked to the corresponding localization, velocity, and distance run. Data were visualized by converting them into a kml file (Keyhole Markup Language) and by the use of Google Earth (Figure 1).

In addition to the PM measurement, a camera was recording the surroundings of the car for further identifications of potential PM sources at concentration peaks of PM.

### 2.2. Driving Routes

In our experimental set up, two different types of routes were driven. Each of them was focusing on certain problems. Local differences in PM concentration and particle size distribution in the driver’s cabin were analyzed on a 15 km circular drive through Frankfurt am. PM concentration differences depending on the ventilation mode (opened window vs. closed window) were quantified on a circular drive in a residential area.

#### 2.2.1. In-Cabin PM Concentration (Local Differences in Concentration and Particle Size Distribution)

**Figure 2:**
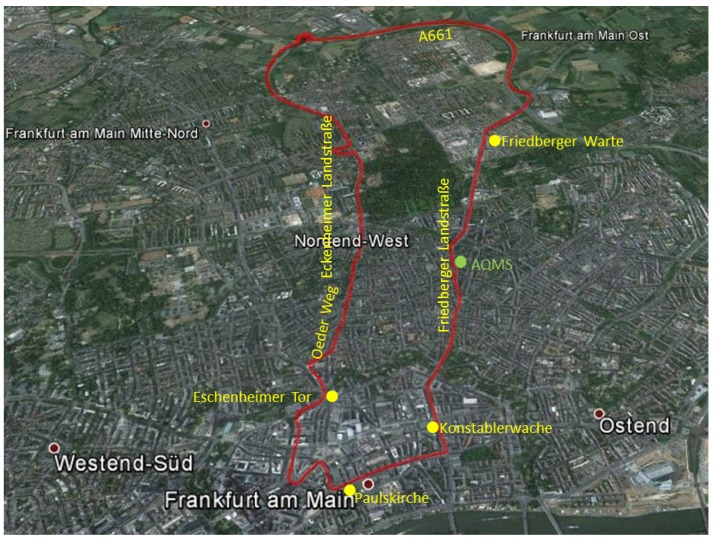
Course 1: Circular drive through Frankurt am Main passing the air quality monitoring stations (AQMS) “Friedberger Landstraße”. Red line: Driving route, yellow marks: Street names and places, green mark: Air quality monitoring station. Geographical picture by Google Earth.

Local differences in PM concentration and particle size distribution in the driver’s cabin were analyzed on a 15 km circular drive through Frankfurt am Main (Course 1; Figure 2).

To collect representative data of the commuter traffic, the route included minor roads, main roads, and a part of a high way. Furthermore, test rides were run on different days of the week before, during, and after rush hour. At peak times, there were numerous traffic jams within the city, especially at traffic lights. Thus, there is a maximum difference in the round time of 17 min (Table 1), with an average round time of 38 min. The test rides were run with different ventilation modes (opened window/closed window).

During the rides with opened window (OW), the window was halfway down and the air conditioning (AC) was switched off. During rides with closed window (CW), the ventilation of the AC was turned on at half of the power with a temperature of 20 °C. A ventilation break of 3 min was made after every ride with OW.

For comparison of the PM data from mobile measurement with AQMS data, the course was chosen to pass the AQMS “Friedberger Landstraße” located at a high-traffic point in Frankfurt am Main. Open-source PM data from the AQMS “Friedberger Landstraße” were downloaded from the homepage from Hessisches Landesamt für Naturschutz, Umwelt und Geologie (HLNUG).

Course 1 was run repeatedly on nine different days between May 2015 and January 2016. Driving dates, intervals, and the point of time while passing the AQMS are given for Course 1 in Table 1.

#### 2.2.2. In-Cabin PM Concentration—Opened Window versus Closed Window

**Figure 3:**
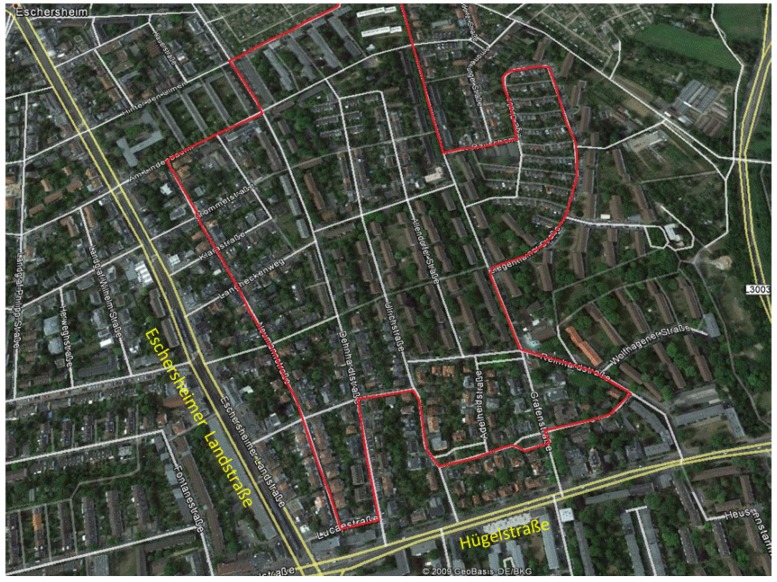
Course 2: Circular drive through a residential area in Frankfurkt-Eschersheim. Red line: Driving route, yellow line: Main roads. Geographical picture by Google Earth.

For better quantification of in-cabin PM concentration differences depending on the ventilation mode (OW vs. CW), a different test location was chosen (Course 2; Figure 3).

In this context, it must be ensured that external conditions, like weather or traffic volume, did not change significantly while concentrations from two following rides were compared with each other. For this purpose, a circular drive in a residential area was chosen. On this much shorter route, with a speed limit of 30 km/h, the traffic volume is very low. The route consisted mainly of one-way streets. Other driving vehicles were only rarely in direct proximity to our test vehicle. The driving time was mostly identical (Table 2). The influence of potential point sources, which lead to high short-term PM concentrations and falsifications in the analyses, were therefore minimized. Nevertheless, the location between two main roads represented the urban traffic influence. The interval between opened window and closed rides was consequently limited to 15 min, so it can be assumed that the background PM concentration did not vary significantly. For the comparison, an alternation between driving with OW and CW was made. During the rides with OW, the window was halfway down and the AC was switched off. During rides with CW, the ventilation of the AC was turned on at half of the power with a temperature of 20 °C. A ventilation break of 3 min was made after every ride with OW. This course was run eight times on each of the four dates. The date and time intervals for the rides in the residential area are listed in Table 2.

### 2.3. Statistics

The local PM concentration and particle size distribution measured inside the cabin were analyzed based on mobile measured PM values with a 6-s resolution.

To discriminate between the short-term concentration peaks of PM and the background concentration, both components must be separated from each other. Percentile-filters were applied on PM values because they eliminate strongly fluctuating short-term concentration peaks, but maintain the slowly changing background concentration. The use of 5%-percentiles (the average of the 5%-lowest values for a certain period) is widely accepted for mobile outdoor measurements [28]. Although the measurements in this study took place inside the cabin, the application of the 5%-percentile seemed to be an adequate method to gain a clear discrimination between both PM trends. For this specific experimental set-up, the velocity driven and the length of the circular drive with an interval of three minutes was used. The 3-min-5%-percentile was computed with *R* and the graphical presentation was made with Graphpad Prism. The coefficient of variation (CV) was calculated as a dimension for the general concentration inhomogeneity measured in the car while driving through the urban area.

For the comparison between the PM concentrations measured inside the car and the PM concentrations measured by the AQMS, two different approaches were applied.

For each test ride on the 15 km circular drive (Course 1), a mean value (PM_mean_) of the mobile measurement was calculated for PM_10_, PM_2.5_, and PM_coarse_. Because the AQMS does not provide data for PM_1_, a comparison in this particle size category was not possible.

Furthermore, PM values of the period of time, when the car passes the AQMS (PM_direct_), were computed for all particle fractions based on the GPS-data and *kml* files. PM_direct_ also is a PM average measured 30 s before and 30 s after the AQMS was passed. Both, PM_mean_ and PM_direct_, were compared to the chronological fitting PM data of the AQMS. A slight deviation could not be avoided because the highest resolution of the AQMS data is given for a 0.5 h mean value. Possible correlations between the PM values from the mobile measurement and the values of the AQMS were analyzed with the Spearman-test and *p*-values were calculated. Additionally, the quotients, PM_mean_/PM_AQMS_ and PM_direct_/PM_AQMS_, were calculated. These ratios reveal PM concentration differences and show whether the mobile measured values relate to the AQMS values. In combination with the corresponding coefficients of variation, the ratios show whether a statement about the driver’s PM exposure based on AQMS measurements can be made. For the analyses of single point sources, size particles were grouped in the categories of 2.5–10 µm, 1–2.5 µm, and <1 µm. For the calculation of the range of particles with diameters between 2.5 and 10 µm, the PM_2.5_ concentration was subtracted from the PM_10_ concentration. For the range of particles with a diameter between 1 and 2.5 µm, the PM_1_ concentration was subtracted from the PM_2.5_ concentration.

In order to compare in-cabin PM concentrations from OW rides with CW rides (Course 2; Figure 3), the median for each test ride was calculated. The medians of the test rides were compared with each other in a rank-based Mann-Whitney U-Test and *p*-values were calculated. In this case, the medians were used as robust values to minimize the influence of short-term concentration peaks, which are typical for single point sources. Particles have been grouped in the categories of 2.5–10 µm, 1–2.5 µm, and <1 µm. The medians of every consecutive opened window-closed window ride were analyzed and compared. The PM reduction achieved by closing the window was calculated separately for each fraction. Finally, the average PM reduction after closing the window was made for each of the four driving days.

## 3. Results

### 3.1. Local Variations of In-Cabin PM Concentrations and Particle Size Distribution

**Figure 4:**
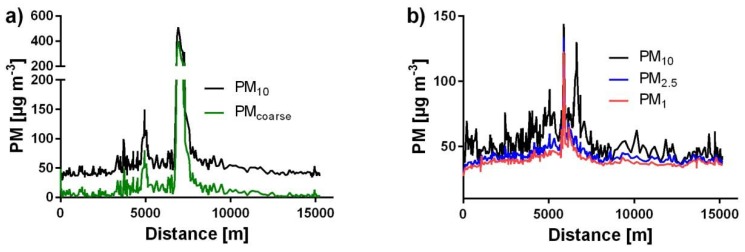
Representative short-term concentration peaks measured with an opened window on 10-20-15. (**a**) At noon, (**b**) in the afternoon.

During the circular drive on Course 1 (Figure 2), maximum OW-concentrations of 508.9 µg m^−3^ for PM_10_ (Figure 4a), 401.3 µg m^−3^ for PM_coarse_ (Figure 4a), 133.9 µg m^−3^ for PM_2.5_ (Figure 4b), and 122.9 µg m^−3^ for PM_1_ (Figure 4b) were measured. Figure 4 shows examples for significantly high concentrations measured during the whole period of this study with a subdivision in different particle size fractions. 

Those short-term concentration peaks generally lasted only seconds or a few minutes. The particle spectrums of these peaks show strong variations. While driving with CW, the concentration pattern was similar, but with lower values of 85.1 µg m^−3^ for PM_10_, 30.3 µg m^−3^ for PM_coarse_, 85.0 µg m^−3^ for PM_2.5_, and 84.6 µg m^−3^ for PM_1_.

**Figure 5:**
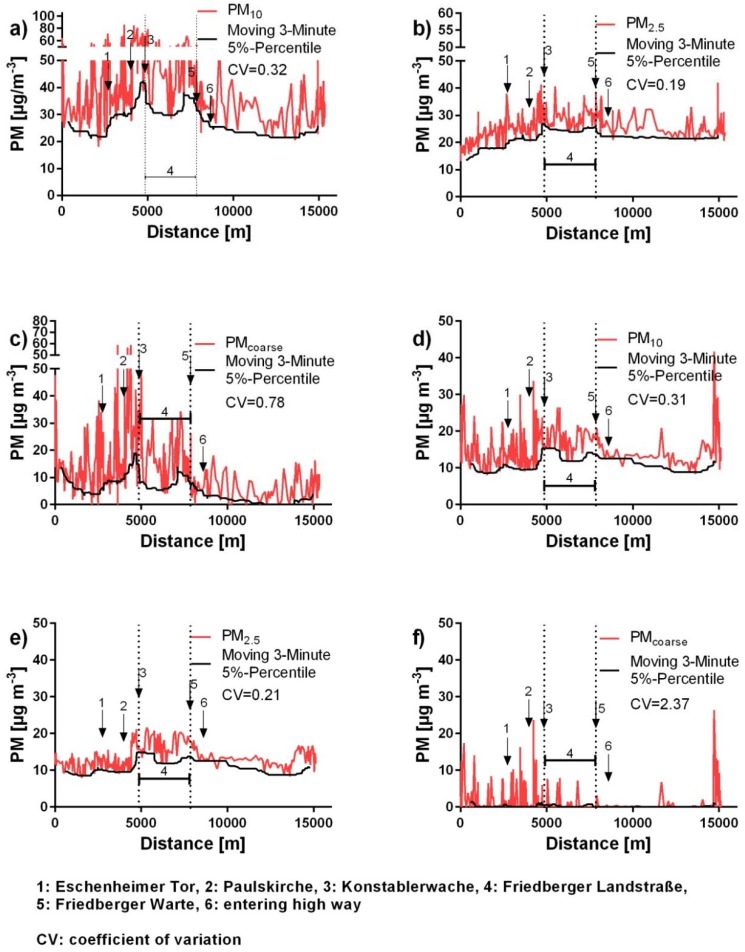
Representative in-cabin PM concentrations with corresponding background concentrations for PM_10_, PM_2.5_, and PM_coarse_ on 01-06-16. (**a**–**c**) Measured while driving with an opened window; (**d**–**f**) measured while driving with a closed window. Geographical positions are marked by the numbers, 1–6.

By visualizing the alternation of the local PM background concentrations measured in the car while driving within the city, characteristic trends were observed (Figure 5).

The PM_10_ background concentrations under OW conditions are slightly changing during the circular drive. The highest concentrations are mainly measured in the city center and the area of “Friedberger Landstraße”. The signal of the absolute PM_10_ concentration shows numerous short-term peaks.

The pattern of the background concentrations of PM_2.5_ and PM_1_ show an increase in the beginning and subsequently stay on approximately the same level. The absolute concentration levels reveal several short-term peaks, but less in frequency and magnitude, than in the PM_10_ signal.

The pattern of the background concentrations of PM_coarse_ vary strongly. The highest concentrations under opened window conditions are mainly measured in the city center and in the area of “Friedberger Landstraße”.

The median coefficient of variation of all rides with OW is 0.32 for PM_10_, 0.20 for PM_2.5_, 0.18 for PM_1_, and 1.04 for PM_coarse_ (*n* = 14).

While driving with a closed window, the pattern of the background concentrations of PM_10_, PM_2.5_, and PM_1_ show a similar trend. They stay approximately on the same level and reach their highest concentrations in the city center.

The CW profiles also show several short-term concentration peaks. The profiles of PM_coarse_ do not show any trends. Only isolated concentration peaks were monitored irregularly. A relation to specific locations in the city was not observed. The median coefficients of variation are 0.30 for PM_10_, 0.21 for PM_2.5_, 0.22 for PM_1_, and 3.07 for PM_coarse_ (*n* = 12).

**Figure 6:**
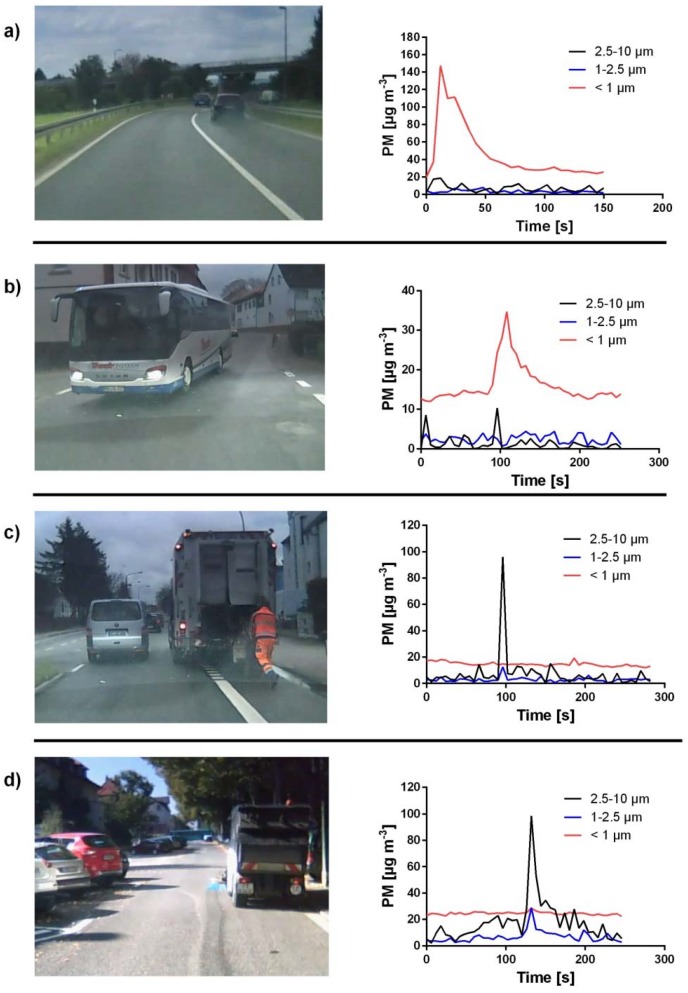
Different PM sources with the corresponding particle size spectrum. (**a**) A car with soot production, (**b**) a bus, (**c**) a garbage truck, (**d**) a street sweeper (see also Supplementary Material 4).

The additional evaluation of the video material allowed the assignment of several short-term concentration peaks to the corresponding sources (Figure 6).

While driving behind a street sweeper or a garbage truck, particles with a diameter between 2.5 and 10 µm dominated. While driving behind a bus or a car with soot production, particles smaller than 1 µm were measured almost exclusively.

These observations were only made while driving with an opened window. In contrast, short-term concentration peaks could not distinctively be connected to any ambient sources while driving with a closed window.

### 3.2. PM In-Cabin versus PM AQMS

Table 3 summarizes the mobile measured in-cabin PM concentrations (PM_mean_, PM_direct_) on the 15 km urban circular drive, Course 1 (Figure 2), with the chronological fitting PM data from the AQMS (PM_AQMS_) for the different particle size fractions and driving modes.

Correlations between PM_mean_ and PM_AQMS_, respectively, between PM_direct_ and PM_AQMS_ are given in Table 4. Rides with OW and rides with CW are analyzed separately.

In-cabin PM_mean_ concentrations correlate significantly with the PM concentrations measured at stationary by the AQMS for the particle fractions, PM_10_ and PM_2.5_. The best correlation between PM_mean_ and PM_AQMS_ was observed for the fraction, PM_2.5_. There was no correlation between PM_mean_ and PM_AQMS_ for the fraction, PM_coarse_. The same observation was made for OW and CW conditions, although the correlation between PM_mean_ and PM_AQMS_ was slightly stronger during an OW-ride.

By comparing PM_direct_ to PM_AQMS_, strong correlations were found for PM_10_ and PM_2.5_. The best correlation between PM_direct_ and PM_AQMS_ was observed for the fraction PM_2.5_. There was no correlation between PM_direct_ and PM_AQMS_ for the fraction, PM_coarse_. The same observation was made for OW as well as for CW. Correlations between PM_direct_ and PM_AQMS_ were slightly stronger for driving with an OW.

While driving with an OW, the correlations between PM_mean_ and PM_AQMS_ were slightly stronger than between PM_direct_ and PM_AQMS_. While driving under CW conditions, the correlation between PM_mean_ and PM_AQMS_ and between PM_direct_ and PM_AQMS_ were on a similar level.

The quotients of the concentrations measured in the cabin (PM_mean_, PM_direct_) and the corresponding data of the AQMS are given in Table 5.

The values of the PM_in car_/PM_AQMS_-quotients for PM_mean_ and for PM_direct_ have a similar magnitude. The quotient is continuously higher than 1.0 while driving under OW conditions, thus concentrations measured inside the car are higher than concentrations measured by the AQMS. The most striking difference between the concentrations measured in the car and those measured by the AQMS can be observed in the PM_2.5_ fraction. While driving under CW conditions, values for PM_2.5_ and, especially, for PM_10_ of the in-cabin measurement are similar to the concentrations measured by the AQMS. The PM_2.5_ fraction still shows slightly higher concentrations inside the cabin in contrast to the AQMS. Particles of the PM_coarse_ fraction show a much lower concentration inside the car in comparison to the concentration measured by the AQMS. High coefficients of variation, especially for PM_coarse_, show that PM_mean_/PM_AQMS_ and PM_direct_/PM_AQMS_ vary strongly between the single rides.

### 3.3. In-Cabin PM Concentration—Opened Window versus Closed Window

**Figure 7:**
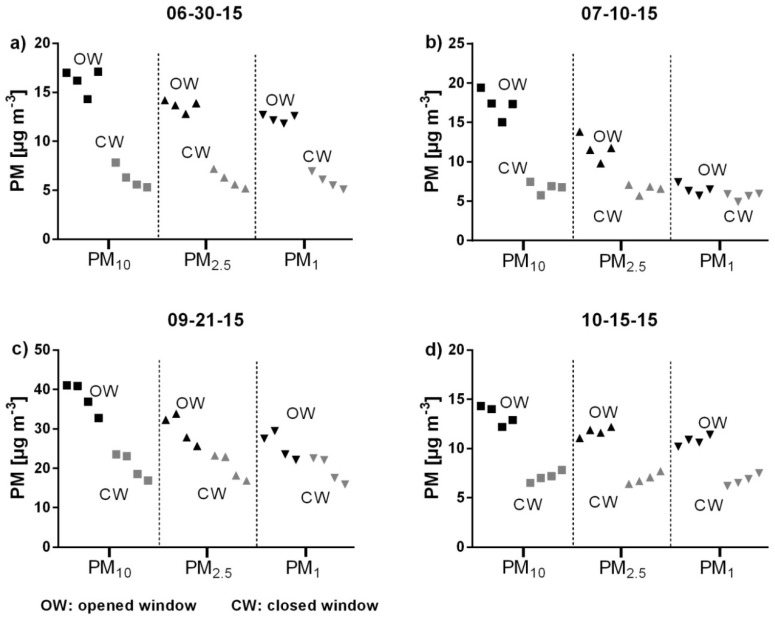
Medians of in-cabin PM_10_, PM_2.5_, and PM_1_ concentrations measured with an opened and closed window. (**a**) On 06-30-15, (**b**) on 01-10-15, (**c**) on 09-21-15, (**d**) on 10-15-15 (see also Supplementary Material 3).

Medians for the PM_10_ concentration range between 12.2 µg m^−3^ and 41.1 µg m^−3^ (OW) and between 5.3 µg m^−3^ and 23.5 µg m^−3^ (CW). For PM_2.5_, medians range between 9.8 µg m^−3^ and 33.8 µg m^−3^ (OW) and between 5.2 µg m^−3^ and 23.2 µg m^−3^ (CW). Medians for PM_1_ range between 5.7 µg m^−3^ and 29.5 µg m^−3^ (OW) and between 4.9 µg m^−3^ and 22.6 µg m^−3^ (CW). Medians of the PM concentration of different particle fractions of four different days are shown in Figure 7.

**Figure 8:**
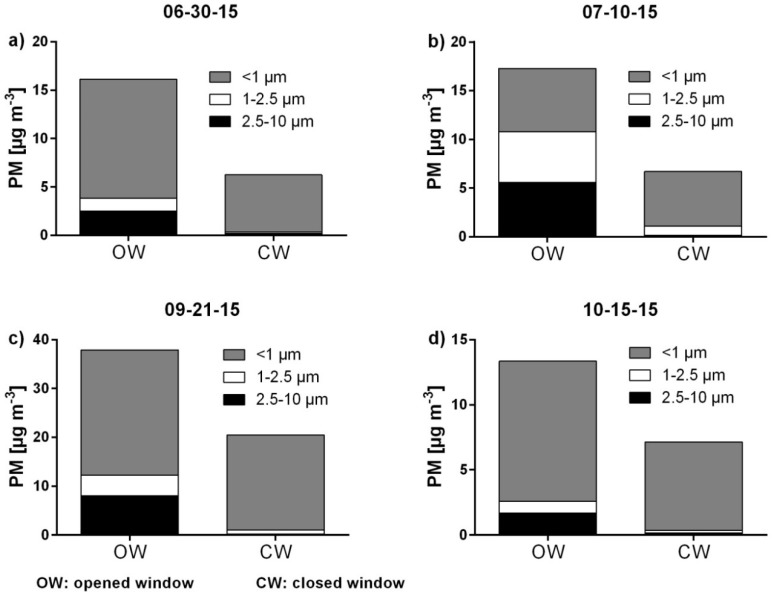
In-cabin PM concentrations and particle size distributions measured with an opened and closed window. (**a**) On 06-30-15, (**b**) on 07-10-15, (**c**) on 09-21-15, (**d**) on 10-15-15.

On each of the four evaluation days, the concentrations of all fractions inside of the car were higher while driving with OW than while driving with CW. Differences in PM concentration could also be observed between the individual test rides (OW as well as CW rides) within a day. Both an increase and a decrease in concentration occurred. The u-test shows significantly high differences between driving with OW and with CW for all fractions with *p*-values <0.0001. The amount of PM reduction differs strongly between the single particle size fractions. In the coarse fraction (2.5–10 µm), there is a relative PM reduction between 87.9% and 97.4% (median: 96.9%) after closing the window. In the next finer fraction (1–2.5 µm), the PM reduction decreases to values between 77.9% and 88.2% (median: 82.3%). In the finest fraction (<1 µm), the PM reduction alternates very strongly between values from 13.0% to 52.0% (median: 31.1%). The absolute PM reduction varies between 1.5 µg m^−3^ and 7.8 µg m^−3^ (median: 4.2 µg m^−3^) for the coarse fraction, between 0.7 µg m^−3^ and 4.3 µg m^−3^ (median: 2.0 µg m^−3^) for the next finer fraction, and between 0.9 µg m^−3^ and 6.4 µg m^−3^ (median: 4.7 µg m^−3^) for the finest fraction. Thus, closing the window has a significant reducing impact on the PM concentrations in the car, especially on the coarser particles (Figure 8).

## 4. Discussion

### 4.1. Variations in the Local Particle Concentration and Size Distribution within the Urban Area

Outside PM concentration and particle size distribution has a significant effect to the concentration measured inside the cabin and is strongly dependent on the location. Even single point sources can lead to concentration shifts inside the cabin [29].

It becomes apparent that in-cabin PM concentrations of the PM_2.5_ and PM_1_ fraction are only changing slightly while driving through the city under OW conditions, with marginal higher values in the city center and on busy roads. These finer particles have a high residence time in the atmosphere and are widely and equally spread. Particles of those fractions can still be measured far away from their sources and therefore can also affect areas with a low primary PM emission within the city [30]. That leads to the comparatively steady in-cabin PM concentration.

Particles in this size range (PM_2.5_, PM_1_) typically originate from vehicle emissions, like abrasion of tires, brakes, and gear shifts, but also from combustion processes. Furthermore, particles from transformation processes of gaseous species and aged particles of other origins occur within this size range [1,30]. Although diesel cars in environmental zones, like in Frankfurt am Main, need to have particle filters and gasoline cars have modern catalytic converters, direct exhaust PM emissions can be measured. Most particles emitted by diesel engines are smaller than 1 µm and mainly appear in two main size ranges. Larger particles with a diameter up to 0.5 µm belong to the accumulation mode and mainly consist of solid material, such as carbon or metallic ash. The smaller particles with diameters under 50 nm belong to the nucleation mode and mainly comprise volatile material [31]. Most particles, especially from the accumulation mode, which partly are in the measuring range of the spectrometer (>0.25 µm), are eliminated by the particle filters. However, there is still a certain part of particles that is not eliminated, especially when the filter is not working probably [32].

In Figure 6a,b, examples for obvious PM sources as direct exhaust emissions are shown. There was probably a problem with the engine or the exhaust system in the car with the visible soot production, whereas behind older buses and heavy duty vehicles, generally high concentrations can be measured as well [21,33]. Also, cars with gasoline engines can emit particles in the measuring range of the spectrometer. Since controlled catalytic converters have been established in Germany since the late 1980s, PM concentrations of emissions of gasoline engines and their particle diameters are low [34,35]. However, especially modern cars with low CO_2_ emissions and direct injection engines emit high PM concentrations with higher particle diameters, which also can be measured by the spectrometer used in this campaign [36]. There were a lot more of those short-term concentration peaks in the size fraction smaller than 1 µm, which could not be linked to certain sources. It can be assumed that several of those peaks also reflect PM emissions from combustion processes of diesel or gasoline cars. The fact that a very high peak concentration for PM_1_ with a value of 122.9 µg m^−3^ was measured while standing at a stop light is an indication for such exhaust-related PM emissions.

In summary, the PM_2.5_ and PM_1_ signal seemed to be a combination of the urban background concentration, mainly of aged mixed aerosols, and the local emission peaks in areas with high traffic caused by abrasion processes and partly by direct exhaust emissions too. The PM fractions, PM_2.5_ and PM_1_, are only limited as being suitable as tracers for traffic emissions because of the relative high background level and its equal distribution. Chowdhury et al. already showed that in urban areas, direct emissions caused by traffic only play a minor role for the ambient PM_2.5_ concentration [37].

Background concentrations of the coarse particles (PM_coarse_) while driving with an opened window was alternating stronger than the background concentration of the smaller fractions. The highest PM levels were reached in the city center, where plenty of short-term peaks can be observed. PM_coarse_ is mainly produced by abrasion of tires or brakes, raised dust, and resuspension in the wake of passing traffic [1]. These coarse particles are eliminated from the atmosphere quickly by depositing [38]. They can only be measured locally. For that reason, they show a strong alternating background signal with many significant short-term concentration peaks. Especially in busy streets, this background value was high in each ride.

Figure 6c,d are typical examples for sources of PM_coarse_. The street sweeper as well as the garbage truck raise dust off the street while cleaning or while depositing garbage.

The PM_10_ signal, which includes the particles of all size categories (PM_1_, PM_2.5_, PM_coarse_), shows a pattern of a rather constant concentration of the finer fractions (PM_1_, PM_2.5_) and also the impact of the strong concentration variations in the coarse fraction (PM_coarse_).

While driving with a closed window, the patterns of PM_10_, PM_2.5_, and PM_1_ were very similar to each other. The reason for that is that almost exclusively, particles smaller than 1 µm were measured. In this case, the PM_1_ concentration matches the PM_2.5_ and the PM_10_ concentrations. Comparing the trend of the PM_2.5_ and the PM_1_ concentration from driving with OW and CW, the highest concentrations were measured in corresponding areas, which are mainly the busy roads in the city center. In contrast to driving with OW, the signal of PM_coarse_ neither shows a background nor a specific trend within the city while driving with CW. Nevertheless, there are some short-term concentration peaks irregularly distributed over the whole ride. They are probably not reflecting the outside concentration. More likely, they display processes inside the cabin, such as raising dust from the seats or the dashboard.

### 4.2. Comparability of Stationary versus Mobile Measured Particulate Matter

Positive correlations for certain particle fractions have shown that PM_mean_ and PM_AQMS_ correlate, even though the mobile measured local changing PM concentrations within a single round are not reflected by the static measured AQMS data. Also, PM_direct_ and PM_AQMS_ concentrations correlate well although a single measured value of the AQMS represents the average concentrations of half an hour. In that time period, traffic may change significantly at the AQMS. Especially, the PM_2.5_ fraction can easily be correlated. That confirms that this fraction is equally distributed in the city and is independent of the location and the time of the measurement. These observations were made during the opened window and closed window rides.

In contrast, the mobile measured PM_mean_ and PM_direct_ concentrations of the fraction, PM_coarse_, are not reflected by the AQMS data, neither while driving with OW nor with CW. Particles of this fraction are distributed irregularly over the whole city, and concentration peaks in general only last for a short period of time. In this case, AQMS data from a single location has no informative value for the real drivers’ exposure.

The analysis of the PM_car_/PM_station_-quotients shows strong alternations between all rides. Developing a constant correction factor to approximate the in-cabin PM concentration from AQMS data is not reasonable.

While driving with OW, the in-cabin PM concentrations were approximately doubled if compared to corresponding values from the AQMS for all particle size fractions. That might be due to different reasons. Our mobile measuring device and thus also passengers in a car are normally much closer to the potential PM sources, like preceding vehicles or raised dust from the street, compared to the sample inlet from the AQMS installed at a height of 3.50 m, with a certain distance to the street [39,40]. Furthermore, the volume of the cabin is very limited so that PM can be accumulated and higher concentrations can be reached [41]. Different studies have already demonstrated that data from AQMS and the actual exposure in a car can differ significantly [18,29,41]. Kaur et al. has found that traffic participants are exposed to generally higher concentrations than data from AQMS would suggest [18]. Praml and Schierl even revealed a three to five times higher PM concentration inside the cabins of buses and trams in contrast to the data from an AQMS station near the road [29].

While driving with CW, concentrations from the mobile measurement and the AQMS are in the same range for PM_10_ and PM_2.5_. Particles of PM_coarse_ reach a much lower concentration in the cabin. So, closing the window and driving with AC on medium ventilation can probably partly reduce the in-cabin PM concentration.

Studies that prove whether in-cabin PM concentrations, generally, are higher than the direct ambient air are discussed controversially [22,42,43,44]. Putative conflicting results can partially be explained by different driving settings. Ventilation mode, traffic situation, and the type of car seem to be critical factors [45,46,47,48].

This study was performed in terms of modern mobility with an electric vehicle. It can be assumed that in vehicles, which themselves have very high PM emissions, even higher in-cabin concentrations can be measured.

For the evaluation, whether the achieved in-cabin PM concentrations have a potential harmful effect to health, limit values have to be set. Because official valid PM limit values are only defined for outside air, results of medical studies for in-cabin PM concentrations can be used for an assessment [49]. Riediker et al. revealed that the average PM_2.5_ concentration of 24 µg m^−3^ reached in the cabin during a shift of highway patrols on four subsequent days led to decreased lymphocytes and increased red blood cells, heart beat cycle length, and heart rate variability [50]. PM values of this magnitude were reached at the mobile OW measurement more often than the AQMS data suspected. Furthermore, high short-term in-cabin PM concentrations with a high medical relevance cannot be detected by the AQMS. The limited room of a cabin favors long-lasting high PM concentrations [41].

The data of the AQMS station can only, to a limited extent, approximate the actual PM exposure of a passenger. The daily shifting background concentration, which is reflected in the AQMS and in-cabin measurements, is mainly responsible for good correlations between AQMS and in-cabin PM concentration values. Meteorological variables, like wind speed or atmospheric stability, are the determining parameters in this case [51]. The daily traffic volume, which underlays high fluctuations, and the different point sources, which appear within one ride irregularly, are partly overlaid by this shifting background concentration. Despite good correlations between AQMS and in-cabin PM concentration values, absolute values strongly differ and thus, an exposure assessment including a medical risk assessment on the basis of AQMS data is highly uncertain.

Especially, high short-term in-cabin exposures cannot be captured by AQMS data and we conclude that mobile measurements are indispensable for monitoring them. Also, the particle size distribution of the PM exposure cannot be analyzed with AQMS data probably because there is no further differentiation within particle sizes smaller than 2.5 µm. The aerosol spectrometer used in our campaign is able to analyze and categories particles down to a size of 0.25 µm. This additional classification is of high medical importance because different particle sizes have different adverse health effects. Especially, airborne particles with aerodynamic diameters less than 1 µm have a high probability of deposition in the respiratory tract. Exacerbate respiratory diseases may be consequences. Small particles also lead to a higher burden of toxins. While absorbing those particles, negative respiratory health effects are possible [52,53,54].

### 4.3. Comparison of In-Cabin PM Exposure on Drivers between Rides with an Opened and Closed Window

When driving with CW and AC turned on, the PM concentration is significantly lower than while driving with an OW. Due to the filtering system and a smaller degree of air exchange, a decrease of the concentration can be reached. Jain and Apte et al. also showed that using AC in a car with CW can lead to lower concentrations while driving through urban areas [33,55]. Closing the window and using the AC also leads to a shift in the particle size distribution to particles with smaller diameters. The car filtering systems are rather suitable for reducing bigger particles. Smaller particles are not affected and not eliminated properly [44].

While driving through areas with a high PM pollution, it is sensible to close the window and use the AC. Thus, the total PM amount is reduced significantly. Although the potentially more harmful particles with smaller diameters are only reduced slightly, on days with a high pollution, also a significant absolute reduction in this size fraction can be reached.

Our test vehicle had a new pollen filter and was parked in an underground car park between the individual test rides. It is expected that the filter performance would decrease after a long run and permanent weather exposure. It must be ensured that vehicles are maintained regularly.

For further PM protection, using the internal recirculation mode from the AC or installing active charcoal filters may be an option [47,56].

Despite the short time frame of a measurement day, the PM concentration differences between the single OW rides and between the single CW rides can be explained by changes in external and internal conditions. Individual point sources along the roadside, such as raised dust or changes in wind speed, are examples of this. Small air turbulences, accumulation processes, or sedimentation can also lead to changes of in-cabin PM concentration.

### 4.4. Limitation of the Study

The PM measurements were carried out by a mobile aerosol spectrometer. Although more precise methods are available, they would not be suitable for mobile measurements. Particles with aerodynamic diameters below 0.25 µm cannot be detected. Since particles with diameters up to 0.1 µm still belong to the particulate matter fraction, a small proportion cannot be measured.

For the comparison of mobile measured data with PM data from stationary measurement, in-cabin concentrations were compared to the chronological fitting PM data of the AQMS. Since the highest resolution of the AQMS data is given for a 0.5 h mean value, slight deviations to the mobile measurement could not be avoided.

## 5. Conclusions

This study showed the importance of mobile measurements in the context of quantifications of in-cabin PM exposure. PM concentrations and PM size distributions were analyzed while driving through the city. Concentrations of smaller particles (PM_2.5_ and PM_1_) had a rather equal level in the city with slightly higher values in areas with a high traffic density. The concentration of coarse particles was rather dependent on single point sources and not distributed evenly over the whole city. Highest concentrations were reached in high-traffic areas. Several concentration peaks with characteristic particle sizes were linked to their sources. The PM_10_ concentration seemed to be the best indicator for traffic-based PM pollution. Smaller particles that show relatively constant background concentrations and emissions of combustion processes on the one hand and coarse particles, which are an indication for areas with a high traffic density in general, on the other hand are included in this particle size fraction.

Data of the AQMS can be used as an indication for the PM exposure to the passengers, but with comparatively low evidence. Especially, the in-cabin concentration while driving with OW would be underestimated and very high short-term concentration peaks cannot be identified. These concentrations measured in the car are of crucial medical relevance. By closing the window and using the AC, the in-cabin PM concentration was significantly reduced. Especially, coarse particles were efficiently eliminated. On the contrary, the elimination of PM with diameters smaller than 1 µm was modest.

It can be assumed that increased particulate matter pollution in urban areas with its health consequences will remain a serious problem in the future. All the more, it is important to consider different exposure scenarios in a differentiated way. Policymakers should also focus on indoor concentrations and, where appropriate, modify limit values.

A worthy extension of our campaign would be the use of aerosol spectrometers for in-depth investigation of the size distribution of ultrafine- and nanoparticles, which are of utmost importance for human health. In addition, a chemical analysis of this small-sized PM may further help to better understand potential health risks from traffic-borne air pollution.

Since, in many places, AQMS data is used for urban risk assessments, a study extension to other cities is reasonable. It is important that the geographical position of the AQMS within the city must be considered individually. Here, ensuring continuous data collection is essential. For a realistic measurement scenario, a representative route should be chosen.

## Figures and Tables

**Table 1:** Test rides on the circular drive through Frankfurt am Main.

(a)				(b)			
OW		CW		OW		CW	
Date	Time	Date	Time	Date	Time	Date	Time
05-22-15	11:59–12:39	05-22-15	11:11–11:52	05-22-15	12:26	05-22-15	11:36
05-22-15	14:26–15:09	05-22-15	13:42–14:23	05-22-15	14:53	05-22-15	14:07
05-29-15	12:42–13:18	05-29-15	12:03–12:39	05-29-15	13:05	05-29-15	12:25
05-29-15	15:50–16:39	05-29-15	15:00–15:45	05-29-15	16:18	05-29-15	15:22
10-15-15	14:57–15:32	10-20-15	11:20–11:54	10-15-15	15:17	10-20-15	11:40
10-20-15	10:42–11:14	10-20-15	14:50–15:23	10-20-15	10:59	10-20-15	15:08
10-20-15	11:57–12:32	12-01-15	11:56–12:32	10-20-15	12:17	12-01-15	13:17
10-20-15	14:09–14:45	12-01-15	17:36–18:13	10-20-15	14:31	12-01-15	18:58
12-01-15	11:16–11:51	12-02-15	15:09–15:50	12-01-15	12:37	12-02-15	16:31
12-01-15	16:47–17:32	01-06-16	15:38–16:12	12-01-15	18:11	01-06-16	15:59
12-02-15	09:26–10:00	01-07-16	15:55–15:27	12-02-15	10:47	01-07-16	11:48
01-06-16	14:55–15:33	01-13-16	18:17–18:57	01-06-16	12:47	01-07-16	15:13
01-07-16	14:17–14:50			01-06-16	15:16	01-13-16	14:52
01-13-16	17:31–18:10			01-07-16	14:35	01-13-16	19:41
				01-13-16	17:52		

(a) Dates with the time and duration of the certain rounds; (b) dates and the point of time when the air quality monitoring stations (AQMS) “Friedberger Landstraße” was passed by the car; OW: Opened window; CW: Closed window.

**Table 2:** Dates and time frames of the test rides through the residential area in Frankfurt-Eschersheim.

	Time			Time	
Date	OW	CW	Date	OW	CW
06-30-15	16:21–16:31	16:34–16:44	09-21-15	10:55–11:04	11:07–11:17
	16:46–16:56	16:59–17:09		11:18–11:28	11:31–11:41
	17:10–17:20	17:24–17:33		11:42–11:52	11:55–12:05
	17:34–17:44	17:48–17:58		12:07–12:17	12:20–12:32
07-10-15	10:52–11:02	11:05–11:15	10-15-15	11:48–11:57	12:00–12:09
	11:28–11:38	11:41–11:50		12:10–12:19	12:24–12:34
	11:53–12:02	12:05–12:15		12:35–12:45	12:48–12:57
	12:23–12:33	12:36–12:46		12:58–13:08	13:11–13:20

OW: Opened window; CW: Closed window.

**Table 3:** Minimum and maximum concentrations for different PM fractions and AQMS data.

			In-Cabin [µg m^−3^]		PM_AQMS_ [µg m^−3^]	
			Min	Max	Min	Max
OW	PM_mean_	PM_10_	17.1	63.7	6.4	35.7
		PM_2.5_	12.9	43.2	3.1	26.6
		PM_1_	10.6	39.5	-	-
		PM_coarse_	3.6	21.6	2.1	9.0
	PM_direct_	PM_10_	18.1	75.7	6.4	36.7
		PM_2.5_	13.5	62.7	3.6	27.3
		PM_1_	11.5	56.7	-	-
		PM_coarse_	1.7	21.7	1.9	9.4
CW	PM_mean_	PM_10_	4.9	27.5	6.3	33.5
		PM_2.5_	4.5	26.4	3.4	24.6
		PM_1_	4.2	25.9	-	-
		PM_coarse_	0.2	1.9	2.0	8.9
	PM_direct_	PM_10_	5.3	26.5	5.5	33.5
		PM_2.5_	5.1	26.4	5.0	24.6
		PM_1_	4.9	25.8	-	-
		PM_coarse_	0.0	2.5	0.5	8.9

Minimum and maximum in-cabin concentrations for the fractions PM_10_, PM_2.5_, PM_1_, and PM_coarse_ and the data of the AQMS measured at approximately the same point of time (see also Supplementary Material 1 and 2). OW: Opened window; CW: Closed window.

**Table 4:** Correlation coefficients for in-cabin PM concentrations and PM concentrations from the AQMS.

		PM_mean_ vs. PM_AQMS_		PM_direct_ vs. PM_AQMS_	
		Spearman	*p*-Value	Spearman	*p*-Value
OW	PM_10_	0.88	<0.001	0.67	0.008
	PM_2.5_	0.93	<0.001	0.82	0.001
	PM_coarse_	0.29	0.31	−0.21	0.46
CW	PM_10_	0.81	0.002	0.78	0.001
	PM_2.5_	0.85	<0.001	0.88	<0.001
	PM_coarse_	−0.14	0.67	−0.21	0.47

OW: Opened window; CW: Closed window.

**Table 5:** Mean PM_mean_/PM_AQMS_-and PM_direct_/PM_AQMS_-quotients of all rides on the circular drive.

		PM_mean_/PM_AQMS_ (Means of All Rides)	PM_direct_/PM_AQMS_ (Means of All Rides)
OW	PM_10_	2.2 (CV: 0.29)	2.2 (CV: 0.46)
	PM_2.5_	2.5 (CV: 0.38)	2.7 (CV: 0.43)
	PM_coarse_	2.0 (CV: 0.68)	1.7 (CV: 0.80)
CW	PM_10_	1.0 (CV: 0.28)	0.9 (CV: 0.20)
	PM_2.5_	1.4 (CV: 0.26)	1.3 (CV: 0.21)
	PM_coarse_	0.2 (CV: 0.82)	0.1 (CV: 1.23)

OW: Opened window; CW: Closed window; CV: Coefficient of variation.

## Supplementary Materials

The following are available online at http://www.mdpi.com/1660-4601/15/12/2642/s1. Data set 1: PM data from the circular drive (mean values) and AQMS data. Data set 2: PM data from the circular drive (direct measurement) and AQMS data. Data set 3: PM data from the residental area. Data set 4:PM data from different point sources.
